# Modulation of mitochondrial function and morphology by interaction of Omi/HtrA2 with the mitochondrial fusion factor OPA1

**DOI:** 10.1016/j.yexcr.2010.01.005

**Published:** 2010-04-15

**Authors:** Nicole Kieper, Kira M. Holmström, Dalila Ciceri, Fabienne C. Fiesel, Hartwig Wolburg, Elena Ziviani, Alexander J. Whitworth, L. Miguel Martins, Philipp J. Kahle, Rejko Krüger

**Affiliations:** a Center of Neurology and Hertie Institute for Clinical Brain Research, 72076 Tübingen, Germany; b Institute of Pathology, University of Tübingen, 72076 Tübingen, Germany; c Medical Research Council Centre for Developmental and Biomedical Genetics, University of Sheffield, Sheffield S10 2TN, UK; d Cell Death Regulation Laboratory, MRC Toxicology Unit, Leicester LE1 9HN, UK

**Keywords:** ANT, adenine nucleotide translocator, Drp1, dynamin-related protein 1, Fis1, mitochondrial fission 1 protein, Hsp90, heat shock protein 90, HtrA2, high temperature requirement protein A2, KO, knockout, MEF, mouse embryonic fibroblast, Mfn2, mitofusin 2, MMP, mitochondrial membrane potential, PBS, phosphate-buffered saline, PD, Parkinson's disease, ROS, reactive oxygen species, SD, standard deviation, SEM, standard error of the mean, VDAC1, voltage dependent anion channel 1, WT, wild-type, Omi, HtrA2, Mitochondria, Fusion, OPA1, Parkinson's disease

## Abstract

Loss of Omi/HtrA2 function leads to nerve cell loss in mouse models and has been linked to neurodegeneration in Parkinson's and Huntington's disease. Omi/HtrA2 is a serine protease released as a pro-apoptotic factor from the mitochondrial intermembrane space into the cytosol. Under physiological conditions, Omi/HtrA2 is thought to be involved in protection against cellular stress, but the cytological and molecular mechanisms are not clear. Omi/HtrA2 deficiency caused an accumulation of reactive oxygen species and reduced mitochondrial membrane potential. In Omi/HtrA2 knockout mouse embryonic fibroblasts, as well as in Omi/HtrA2 silenced human HeLa cells and *Drosophila* S2R+ cells, we found elongated mitochondria by live cell imaging. Electron microscopy confirmed the mitochondrial morphology alterations and showed abnormal cristae structure. Examining the levels of proteins involved in mitochondrial fusion, we found a selective up-regulation of more soluble OPA1 protein. Complementation of knockout cells with wild-type Omi/HtrA2 but not with the protease mutant [S306A]Omi/HtrA2 reversed the mitochondrial elongation phenotype and OPA1 alterations. Finally, co-immunoprecipitation showed direct interaction of Omi/HtrA2 with endogenous OPA1. Thus, we show for the first time a direct effect of loss of Omi/HtrA2 on mitochondrial morphology and demonstrate a novel role of this mitochondrial serine protease in the modulation of OPA1. Our results underscore a critical role of impaired mitochondrial dynamics in neurodegenerative disorders.

## Introduction

Omi/HtrA2 (high temperature requirement protein A2) is a nuclear encoded serine protease that localizes to the mitochondrial intermembrane space, and is released into the cytosol upon apoptosis [1]. The pro-apoptotic function of the Omi/HtrA2 protease is at least partially mediated by binding and proteolytic removal of inhibitor of apoptosis proteins [2-4]. However, recent *in vivo* and *in vitro* data indicate that Omi/HtrA2 has physiological cytoprotective role(s) within the mitochondria of non-apoptotic cells. In fact, loss of Omi/HtrA2 may contribute to selective neurodegeneration [5,6]. Loss of mitochondrial serine protease activity in *mnd2* mice, which is caused by a homozygous point mutation in the Omi/HtrA2 gene, leads to a phenotype with muscular wasting and striatal neurodegeneration [7,8]. Likewise, targeted disruption of the Omi/HtrA2 gene in mice led to progressive, severe striatal neuron loss, consequently resulting in a locomotor phenotype [6]. Interestingly, in striatal neurons of mutant huntingtin overexpressing mice as well as in the affected brain regions of human Huntington's disease patients, reduced levels of Omi/HtrA2 were found [5], supporting the notion that Omi/HtrA2 deficiencies are involved in neurodegeneration. Finally, mutations in the Omi/HtrA2 gene have been implicated as a susceptibility factor in German and Belgian patients with sporadic Parkinson's disease (PD) [9,10].

Recent studies in various neurodegenerative diseases have shown that altered mitochondrial function and dynamics take center stage in neuronal viability [11], particularly in PD [12]. Mitochondria are metabolically active and highly dynamic organelles that constantly undergo fusion and fission events in order to maintain integrity. This has not only implications for mitochondrial morphology, but the control of these antagonistic activities is directly linked to mitochondrial function [13,14]. Due to the specific energy needs of the nervous system and the non-dividing character of the implicated cell type, disturbed mitochondrial dynamics are critical for the accumulation of dysfunctional mitochondria characterized by increased production of reactive oxygen species (ROS), decreased mitochondrial membrane potential and damaged mitochondrial DNA. In this context, proteins that directly modulate mitochondrial fusion have been found mutated in neurodegenerative diseases like Charcot-Marie-Tooth 2A (Mitofusin-2 (Mfn2); [15]) or autosomal-dominant optic nerve atrophy (optic atrophy protein 1 (OPA1); [16,17]). Mutations in the OPA1 protein as well as increased proteolytic processing of OPA1 lead to impaired mitochondrial fusion and dysfunction of the organelle [18,19]. Recently, also other PD associated genes, namely Parkin and PINK1, have been directly linked to mitochondrial homeostasis [20] and morphology [21].

Based on the critical involvement of mitochondrial function in neurodegeneration and aging processes and due to the role of mutations in the Omi/HtrA2 gene as possible susceptibility factors in PD [9,10], we studied the consequences of loss of Omi/HtrA2 protein in fibroblasts from knockout mice [6] as well as in silenced human HeLa and *Drosophila melanogaster* S2R+ cells. We describe for the first time a role of Omi/HtrA2 in the regulation of the key fusion protein OPA1 that is linked to mitochondrial elongation.

## Materials and methods

### Cell lines and culture

Immortalized mouse embryonic fibroblasts (MEFs) from Omi/HtrA2 knockout (KO) mice and wild-type (WT) controls [6] were grown in Dulbecco's modified Eagle's medium (Invitrogen) with added 10% fetal calf serum (Perbio Science) and 1% penicillin/streptomycin (Invitrogen). Both cell lines were kept at the same passage number for experimental consistency.

Stably transfected human embryonic kidney HEK293 cells were generated using pCMV-Tag4 empty vector (Stratagene) or containing human WT Omi/HtrA2-FLAG insert [9]. HEK293 cells were transfected using Fugene6 (Roche), and positive clones selected by the continued addition of 500 μg/ml G-418 (Invitrogen) to the growth medium.

A human cell line derived from cervical cancer (HeLa) was used for silencing experiments. HeLa cells were cultured in Dulbecco's modified Eagle's medium (Invitrogen) with added 10% fetal calf serum (Perbio Science). All cells were incubated in a 5% CO_2_ humidified atmosphere at 37 °C.

*Drosophila* S2R+ cells were cultured in Schneider's medium (Invitrogen) supplemented with 5% fetal calf serum (Sigma) and 1% penicillin/streptomycin (Invitrogen-Gibco).

Stably re-transfected KO MEF cells were generated using human WT or a protease dead mutant (S306A) of Omi/HtrA2 without the FLAG tag (see above) subcloned into the pcDNA 3.1/Zeo vector (Invitrogen) or empty vector as control. The S306A mutant was cloned using the Stratagene QuikChange II Site-Directed Mutagenesis Kit according to manufacturer's instructions. Omi/HtrA2 KO MEF cells were transfected using Fugene6 (Roche), and positive clones selected by the continued addition of 500 μg/ml zeocin (Invivogen) to the growth medium.

### Flow cytometry

For all flow cytometry measurements, the cells were harvested using phosphate-buffered saline (PBS) with 2 mM EDTA and washed once with PBS after which they were stained with appropriate dyes.

To measure levels of cellular reactive oxygen species (ROS), the cells were stained with 10 μM 2′,7′-dichlorohydrofluorescein diacetate (Invitrogen) in PBS for 30 min at 37 °C. The cells were then washed twice with PBS, resuspended in PBS and kept on ice for the measuring procedure. For each sample, ∼ 20,000 cells were measured using the 488 argon laser and emission through the FITC filter (530 nm) of a CyAn flow cytometer (DakoCytomation).

To measure the levels of mitochondrial superoxide, the cells were stained with 5 μM MitoSOX Red (Invitrogen) in PBS for 15 min at 37 °C. The cells were then washed once with PBS and resuspended in PBS. For each sample, ∼ 70,000 cells were measured using the 488 argon laser and emission through the PE filter (575 nm).

To measure the mitochondrial membrane potential, the cells were stained with 100 nm MitoTracker Green FM and MitoTracker CM-H_2_XRos (Invitrogen) in PBS for 15 min at 37 °C. The cells were then washed once with PBS and resuspended in PBS. For each sample, ∼ 70,000 cells were measured using the 488 argon laser and emission through the PE Texas Red filter (613 nm) and the FITC filter (530 nm).

For all flow cytometry measurements, KO MEF cells were compared against WT MEF cells and re-transfected vector controls were compared against WT Omi/HtrA2 re-transfected KO cells. For measurements of mitochondrial ROS, the threshold was set at about 50% of the WT signal intensity and the percentage of shift in the fluorescence activated cell sorter (FACS) staining was determined with the Summit version 4.2 software (DakoCytomation). All the presented results are the means of three independent experiments performed in duplicate and are presented as relative change compared to the WT condition.

### Analysis of mitochondrial morphology

MEF and HeLa cells were stained 10 min at 37 °C with 125 nM MitoTracker Green FM and Hoechst 33342 in DMEM medium (all Invitrogen). The cells were then washed once with PBS and covered with DMEM. For each cell line, pictures were taken from live cells using a Cell Observer (Carl Zeiss) at 37 °C and 5% CO_2_. Each cell was classified into one of the following groups related to the predominant phenotype of mitochondria: elongated, middle sized or fragmented using the same criteria for each picture. For HeLa cells, two categories were applicable for scoring: elongated and normal. A number of 100–150 cells were scored per experiment and each experiment was done at least in triplicate. The scoring was done by an unbiased investigator blinded to the genotype and the treatment of the cells.

For *Drosophila* mitochondrial morphology analysis, live cells were incubated with 200 μM rhodamine 123 and imaged directly in growing medium. Quantification of mitochondria length was performed using the ImageJ software as previously described [22].

### RNA interference

Silencing of Omi/HtrA2 was performed with HiPerformance siRNA (Qiagen), targeting the following sequence of human Omi/HtrA2, 5′-CAGCACCTGCCGTGGTCTATA-3′. A scrambled siRNA was used as control (Qiagen). HeLa cells were transfected with 5 nM siRNA on three consecutive days using HiPerFect (Qiagen) according to manufacturer's instruction. Cells were transferred into chamberslides for immunofluorescence after the third transfection and analyzed 2 days later. Protein expression and silencing were verified by Western blot analysis.

Double stranded RNAs were prepared using MEGA script kit (Ambion). Primers used to generate dsRNAs are available upon request. S2R+ cells were plated (1.2 × 10^6^ per well) in a 6-well plate and treated with 15 μg dsRNA in serum-free medium using Effectene (Qiagen). Two hours after dsRNA treatment, complete medium was added to the wells and cells were cultured for 2 days before being imaged.

### Electron microscopy

Omi/HtrA2 KO and WT MEFs were fixed in 2.5% glutaraldehyde in Hank's modified salt solution (HMSS), postfixed in 1% OsO4 in 0.1 M cacodylate buffer, scraped off, centrifuged and dehydrated in a series of ethanol. The 70% ethanol step was saturated with uranyl acetate for contrast enhancement. Dehydration was completed in propylene oxide and the specimens were embedded in Araldite (Serva). Ultrathin sections were produced on a FCR Reichert Ultracut ultramicrotome (Leica), mounted on pioloform-coated copper grids and contrasted with lead citrate. Specimens were analyzed and documented with an EM 10A electron microscope (Zeiss). Subsequently mitochondria were counted and categorized in 50 cells of each condition.

### Western blot analysis

Cells were harvested with 2 mM EDTA in PBS and lysed with TNE (50 mM Tris–HCl [pH 7.4], 150 mM NaCl, 1 mM EDTA and 10 mM NaPP) containing 1% Triton X-100 (Sigma) and Complete protease inhibitor cocktail (Roche). Protein concentration was measured using Bradford solution (Bio-Rad) in a microplate reader and the protein lysate (20-35 μg) was separated by SDS-PAGE. Alternatively, 150,000 cells were counted, harvested and lysed directly in Laemmli SDS-PAGE sample buffer (62.5 mM Tris–HCl [pH 6.8], 2% sodium dodecyl sulfate (SDS), 5% β-mercaptoethanol, 10% glycerol, 0.01% bromophenol blue) by boiling, before subjecting them to SDS-PAGE. Western blots were analyzed by antibodies against Omi/HtrA2, mitochondrial fission 1 protein (Fis1) (both from Axxora), OPA1 (BD Transduction Laboratories), mitofusin 2 (Mfn2) or β-actin (both from Sigma), adenine nucleotide transporter (ANT) or voltage dependent anion channel 1 (VDAC1) (both from Calbiochem), prohibitin (NeoMarkers), and heat shock protein 90 (Hsp90; Stressgen).

S2R+ cells were harvested in RIPA buffer (50 mM Tris–HCl [pH 7.4], 150 mM NaCl,), supplemented with Complete protease inhibitor cocktail. Protein concentration was measured using Bradford solution (Sigma) and the protein lysate (20–30 μg) was separated by SDS-PAGE, after addition of loading buffer (50 mM Tris–HCl; pH 6.8, 2% SDS, 10% glycerol, 1% β-mercaptoethanol, 12.5 mM EDTA, 0.02% Bromophenol Blue ). Western blots were analyzed using antibodies against Omi/HtrA2 (1:1000, kindly provided by M. Martins) and anti-β-tubulin (1:10000, Sigma). For detection, secondary antibodies conjugated with HRP (Chemicon) were used (1:5000), and immunoreactivity was visualized with ECL chemiluminescence (Amersham).

For densitometric analyses of OPA1 levels on Western blots, the ImageJ software (Rasband, W.S., ImageJ, U.S. National Institutes of Health, USA, http://rsb.info.nih.gov/ij/, 1997–2007) was used. For Fig. 5B, OPA1 levels were normalized to the loading control and further all samples were compared to Omi/HtrA2 WT MEF levels which were set to 100%. The results represent the mean of comparison of three independent experiments. For the fold change calculation in Fig. 7D, the densitometric analysis of the indicated band was performed for each treatment and these were compared to the untreated controls (0 min of both WT and KO).

### Immunoprecipitation

For immunoprecipitation in HEK293 cells stably overexpressing Omi/HtrA2, lysates were made with an immunoprecipitation lysis buffer (50 mM HEPES [pH 7.5], 10 mM KCl, 50 mM NaCl, 1 mM EDTA, 0.5 mM EGTA, 1.5 mM MgCl_2_, 10% glycerol, 0.2% NP-40) and were incubated with anti-FLAG agarose beads (Sigma) overnight at 4 °C. After 5 washes with lysis buffer the samples were analyzed by Western Blot analysis.

For immunoprecipitation from mouse brain, one hemisphere from a C57/Bl6 mouse was homogenized in homogenization buffer (50 mM Tris [pH 7.5], 1% [v/v] NP-40, 2 mM EDTA, 100 mM NaCl and Complete protease inhibitor cocktail). Ten percent NP-40 (Fluka) was added and the mixture incubated on ice for 30 min. To remove cell debris, the lysates were centrifuged twice after which Protein G agarose alone or pre-incubated with OPA1 antibody was added and incubated overnight at 4 °C before it was washed and analyzed by Western blotting.

### RT-PCR

Total mRNA was isolated from MEF cells using peqGOLD TriFast reagent following manufacturer's instructions (Peqlab) and 600 ng total RNA was reverse transcribed using Transcriptor High Fidelity cDNA Synthesis Kit (Roche) and anchored oligo-dT Primer to exclude non-message RNA from reverse transcription. Two microliters of RT reaction was subsequently used as template for transcript amplification in a 25 μl reaction with 5 μl 5× GoTaq Buffer, 0.1 μl GoTaq Polymerase (Promega) and 2 μM specific primers for *OPA1* designed to amplify all isoforms (sense 5′-ACTCAGTTTATGTTCACCAC-3′, antisense 5′-AGAGCACACAATATTCAAAC-3′) and for *β-actin* (sense 5′-GGGTCAGAAGGACTCCTACG-3′, antisense 5′-GGTCTCAAACATGATCTGGG-3′). The annealing temperature was 48 °C for *OPA1* and 52 °C for β*-actin* amplification, respectively. Elongation time was 30 s in both cases. Cycle number was chosen in the linear range of amplification of each transcript (27 cycles for *OPA1* and 20 cycles for β*-actin*), according to the expression level. Amplified PCR products were resolved by 1% agarose gel electrophoresis and stained with an ethidium bromide solution (Carl Roth).

For mRNA quantification in *Drosophila* cells, total RNA was extracted using TRI Reagent (Sigma) or RNeasy Mini Kit (Qiagen) following manufacturer's instruction. Total RNA (1.5 μg) was reverse-transcribed by using a random decamer primer (RETROscript kit, Ambion). Quantitative real-time PCR was performed using the SYBR Green Master Mix method (Sigma) with a Bio-Rad MyiQ system. Primers used are as follows: for *Omi/HtrA2* (sense 5′-GCCCTGGCGGATAATAGTAA-3′ and antisense 5′-GCTGCATACAGGTTAAACTAGGG-3′) and for the housekeeping gene *GAPDH* (sense 5′-GCGAACTGAAACTGAACGAG-3′ and antisense 5′-CCAAATCCGTTAATTCCGAT-3′).

### Mitochondrial isolation and proteinase K digest

Omi/HtrA2 WT and KO cells were grown to 100% confluence on two 15 cm plates, harvested, washed twice with PBS and resuspended in mitochondrial isolation buffer (MIB) (250 mM sucrose, 20 mM HEPES [pH 7.5], 3 mM EDTA and Complete protease inhibitor cocktail). The cells were disrupted by 25 passes with a glass douncer and subsequently 15 passes through a 20 Gauge needle before they were centrifuged at 830 × *g* for 10 min. From this, the supernatant was retained and the pellet resuspended in MIB and disrupted as before, and again centrifuged at 830 × *g* for 10 min. The combined supernatants were further centrifuged at 16,800 × *g* for 10 min. The supernatant was kept as the cytosolic fraction and the pellet washed twice more with MIB before resuspended in 100 μl MIB. The protein concentration was measured using Bradford solution. The purity of the preparation was confirmed by immunoblot analysis. For proteinase K digest, 20 μg mitochondria were subjected to 20 ng proteinase K (Merck, EC 3.4.21.64) digestion at 37 °C for 0, 5, 10, 20, and 40 min. The reaction was stopped with 0.8 μl stop solution (100 mM EDTA, 40 mM phenylmethyl-sulphonyl fluoride) before the mitochondria were spun down at 16,000×*g* for 10 min and resuspended in PAGE sample buffer for immunoblot analysis.

### Statistical analysis

Experimental results for mitochondrial morphology were analyzed for statistical significance using the Student's *t*-test implemented in Microsoft Excel Software. For analysis of the mitochondrial length in *Drosophila* S2R+ cells, the significance was determined by ANOVA with a Bonferroni post hoc correction. For statistical significance of the densitometrically analyzed Western blot experiments, a one-way ANOVA and a Student Newman–Keuls post hoc correction were performed using GraphPad InStat version 3.00 for Windows 95 (GraphPad Software, San Diego California USA, www.graphpad.com).

## Results

### Increased ROS in Omi/HtrA2 KO MEF Cells

**Fig. 1:**
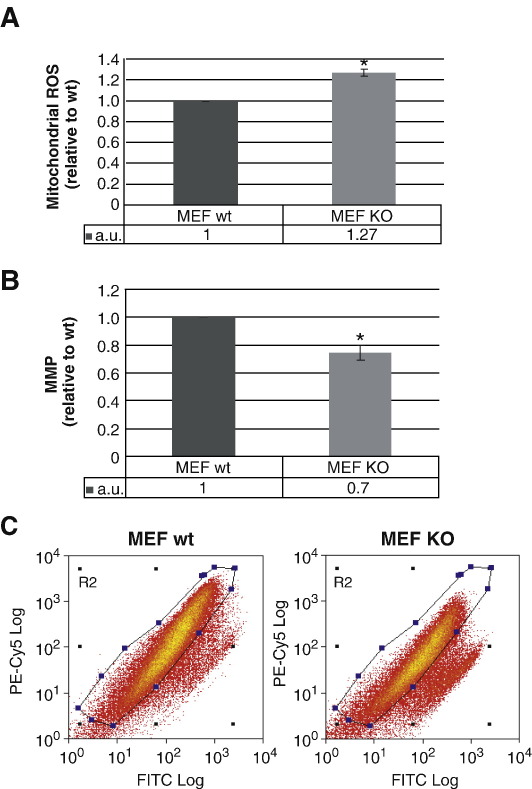
Analysis of ROS levels and mitochondrial membrane potential in Omi/HtrA2 WT and KO MEF cells. (A) Mitochondrial ROS levels were analyzed by staining with the fluorescent dye MitoSOX. (⁎*p* < 0.006). (B, C) MMP was determined by a double staining with MitoTracker GreenFM and MitoTracker CM-H_2_XRos. (B) The ratio of MitoTracker GreenFM to Mitotracker CM-H_2_XRos staining was measured as a readout for the MMP (⁎*p* <   0.003). Error bars show the standard deviation (SD). (C) Scattergraph representative of an Omi/HtrA2 WT (left graph) and KO (right graph) MEF MMP measurement. The selected area in blue represents the measured cells with intact MMP. The loss of signal in the marked area indicates a decrease in MMP. All results are given as relative comparisons to WT. The results represent the mean of three independent experiments.

First we analyzed the effects of Omi/HtrA2 KO on mitochondrial function and integrity. Dysfunction of mitochondria is typically reflected by disturbed oxidative phosphorylation and leads to the generation of ROS. Intramitochondrial ROS production was quantified using MitoSOX Red, a fluorogenic dye that is targeted to the mitochondria and readily oxidized by superoxide, but not by other ROS- or reactive nitrogen species-generating systems [23]. We found a significant increase in intramitochondrial ROS in MEF cells from Omi/HtrA2 KO mice compared to WT controls (Fig. 1A). Moreover, total ROS levels, measured by 2′,7′-dichlorohydrofluorescein diacetate conversion, were elevated. An average 15.2% ± 1.2% increase was observed in the Omi/HtrA2 KO MEFs (*p* = 0.0022) (data not shown). These results demonstrate substantial oxidative stress occurring in MEF cells devoid of Omi/HtrA2, confirming previously published findings that show increased ROS production in immortalized Omi/HtrA2 KO MEF cells [24]. This could be related to decreased respiration, as shown previously for brain mitochondria isolated from Omi/HtrA2 KO mice [24].

### Decreased mitochondrial membrane potential in Omi/HtrA2 KO MEF cells

We next examined the effects of Omi/HtrA2 knockout on the mitochondrial membrane potential (MMP). We measured the MMP by flow cytometry of cells dual-labeled with MitoTracker Green FM and MitoTracker CM-H_2_XRos (Invitrogen). As MitoTracker Green FM labels all mitochondria independently of membrane potential and MitoTracker CM-H_2_XRos is only taken up into mitochondria with an intact membrane potential, a ratio between these two signals allows to determine how many of the cells counted have mitochondria with an intact membrane potential. As this method simultaneously compares mitochondria with an intact membrane potential to all mitochondria, it controls for the loss of mitochondrial mass as a potential source for decreased signal. Omi/HtrA2 KO MEF showed a significant decrease in MitoTracker Red/Green signal compared to WT controls (Figs. 1B, C) indicating MMP decrease in the KO cells. These results were confirmed using tetramethylrhodamine methyl ester as a single dye technique (data not shown), the latter being in line with previous studies [25,26]. The consistent MMP reduction in Omi/HtrA2 KO MEF cells clearly demonstrates that Omi/HtrA2 is necessary for the maintenance of an intact MMP.

### Mitochondrial morphology regulated by Omi/HtrA2

**Fig. 2:**
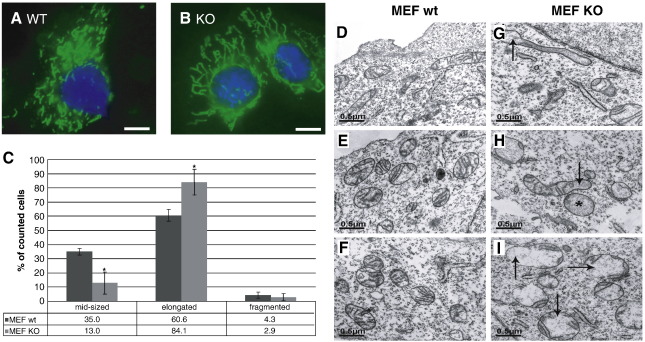
Effects of Omi/HtrA2 on mitochondrial morphology in MEF cells. (A, B) Live Omi/HtrA2 WT (A) and KO (B) MEF cells were concomitantly stained with MitoTracker GreenFM and Hoechst 33342 for 10 min. Size bar corresponds to 10 μm. (C) Mitochondrial morphology was classified into 3 categories: elongated, middle sized and fragmented. The results are shown as the percentage of WT (dark bars) and KO (light bars) cells with mitochondria falling into these categories as scored by an observer blinded to the MEF cell genotype (⁎*p* <   0.05). Error bars show the SD. (D–I) Electron microscopy shows that mitochondria in MEF KO cells (G–I) differed from control MEF WT cells (D–F) by failure to form elaborated cristae structures. Examples of mitochondria with a local loss of cristae structures (arrow in H and I) or a complete loss of folded inner membrane structures (asterisk in H and arrow in G).

Recent data underscore the relevance of mitochondrial dynamics in neurodegeneration. It has been shown that mitochondrial dysfunction leading to free radical generation and loss of energy supply is involved in dynamic morphological alterations of mitochondria [11]. To monitor mitochondrial morphology, we performed fluorescence microscopy based on MitoTracker green and concomitant Hoechst 33342 nuclear staining. This was performed using live cell imaging in order to avoid fixation artifacts. In Omi/HtrA2 KO cells, we found an increased proportion of cells that display sustained, elongated, net-like structure of mitochondria (Figs. 2A–C).

Mitochondrial ultrastructure was analyzed by electron microscopy. Control MEF cells displayed typical round shaped mitochondria with well-formed cristae (Figs. 2D–F). In contrast, Omi/HtrA2 KO cells exhibited a substantial amount of mitochondria with altered morphology (Figs. 2G–I). Semiquantitative analysis revealed that 44.5% of the mitochondria in Omi/HtrA2 KO MEF cells show these alterations while only 8.6% mitochondria in Omi/HtrA2 WT MEFs have abnormal mitochondrial ultrastructure. Typical alterations were bulged, extended mitochondria, often with less inner membrane structures and no observable cristae structure, including a loss of cristae junctions. At many sites, in particular if the mitochondria were cut longitudinally, we observed a local loss of cristae, whereas the other end of the mitochondrium was still normal. If the part with the lost cristae was cross sectioned, the mitochondrium seemed to be completely devoid of folded inner membrane structures. These alterations of mitochondrial ultrastructure are consistent with the elongated morphology seen with light microscopy (Fig. 2).

**Fig. 3:**
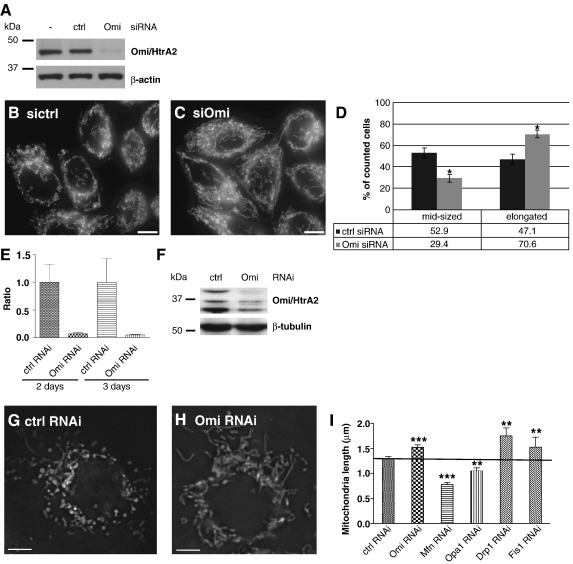
Mitochondrial morphology in Omi/HtrA2 silenced HeLa and S2R+ cells. (A) Treatment of HeLa cells with Omi/HtrA2 siRNA greatly reduced the levels of Omi/HtrA2 protein compared to non-transfected (−) and control siRNA treated cells (ctrl). Re-probing the Western blot for β-actin confirmed equal loading. (B–D) The mitochondrial morphology was investigated in HeLa cells transfected with control siRNA (B) or Omi/HtrA2 siRNA (C). The mitochondria were scored in each cell as either elongated or normal and quantified (D). Error bars show the SD; ⁎*p* <   0.005. Scale bar corresponds to 10 μm. (E) Quantitative real-time PCR measurement of *Omi/HtrA2* knock down in S2R+ cells compared to control. (F) Western blot of Omi/HtrA2 levels in S2R+ control and Omi/HtrA2 dsRNA treated cells. β-Tubulin was used as a loading control. (G–I) S2R+ cells treated with control (ctrl) (G) or *Omi/HtrA2* (H) dsRNAs stained with mitochondrial dye rhodamine123 dye. Scale bars correspond to 5 μm. (I) Mitochondrial length was quantified and compared to knock down of known fission and fusion factors (Mfn/Marf, OPA1, Drp1, Fis1) (⁎⁎* p *< 0.01, ⁎⁎⁎* p *< 0.001). Error bars show the standard error of the mean (SEM).

To control for cell type and species specific effects, we extended our morphological studies to human HeLa cells and *Drosophila* S2R+ cells. Transient silencing of Omi/HtrA2 in HeLa cells using an siRNA approach resulted in a clear loss of the protein (Fig. 3A) and morphologically a similar phenotype as in MEF cells devoid of Omi/HtrA2, namely elongated mitochondria (Figs. 3B–D). Similarly, the transient silencing of Omi/HtrA2 in *Drosophila* S2R+ cells lead to a loss of both Omi/HtrA2 mRNA (Fig. 3E) and protein (Fig. 3F) and an elongation of the mitochondria as measured by the length of the mitochondria (Figs. 3G–I). As a comparison, known fission and fusion genes were also silenced (Fig. 3I). The effect of Omi/HtrA2 silencing on mitochondrial morphology was comparable to the effects of silencing the established fission proteins Fis1 and dynamin-related protein 1 (Drp1) in *Drosophila*. These results clearly underscore a well conserved role of Omi/HtrA2 in regulating mitochondrial morphology.

### Mitochondrial morphology relies on Omi/HtrA2 protease function

**Fig. 4:**
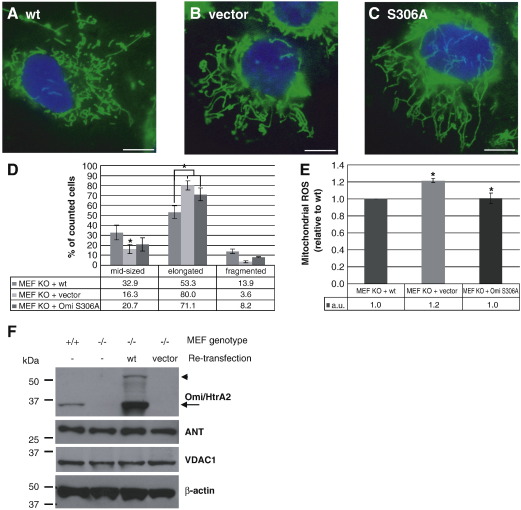
Re-transfection of KO MEFs with human WT Omi/HtrA2 rescues the phenotype. (A–D) The mitochondrial morphology of Omi/HtrA2 KO MEF cells stably overexpressing either human WT Omi/HtrA2 (A), empty vector (B) or a synthetic protease dead mutant of Omi/HtrA2 (C) was investigated using the same procedure as in Fig. 2. Scale bar corresponds to 10 μm. In the quantification (D), error bars indicate the SEM (⁎*p* <   0.05). (E) Stably re-transfected MEF cells were stained with the fluorescent dye MitoSOX to investigate the levels of intramitochondrial ROS. The procedure was the same as was used for the WT and KO cells. The result is the mean of three independent experiments and a relative comparison of Omi/HtrA2 KO re-transfected with vector compared to those re-transfected with WT or S306A Omi/HtrA2, where WT was set to 1 (⁎*p* <   0.06). Error bars indicate the SD. (F) WT and KO MEF cells as well as KO MEFs retransfected with either WT Omi/HtrA2 or empty vector were lysed with 1% Triton X-100 in TNE. The stable overexpression of human Omi/HtrA2 leads to expression of an ≈ 50 kDa precursor (arrow head) that is efficiently processed to the ≈ 35 kDa mature form (arrow) co-migrating with the endogenous Omi/HtrA2, which is completely absent in KO MEF cells and vector controls when analyzed by Western blot. To investigate whether there is a change in the mitochondrial membrane mass, the relative amounts of the outer membrane protein VDAC1 and inner membrane protein ANT were also probed. β-Actin was used as a loading control.

To investigate whether the altered mitochondrial phenotype seen upon loss of Omi/HtrA2 can be reversed and whether it is dependent on the protease function of the protein, we stably transfected KO MEF cells with empty vector or a plasmid expressing human WT Omi/HtrA2 cDNA. In addition, we generated a protease dead mutant [S306A]Omi/HtrA2 [2-4] and produced the corresponding stably re-transfected MEF cell lines. Indeed, re-transfection of WT Omi/HtrA2 (Fig. 4A), but not an empty vector (Fig. 4B), was able to revert the mitochondrial phenotype in Omi/HtrA2 KO MEFs to a similar degree as observed in MEFs from wild-type littermate controls (Fig. 2C). In contrast, the protease dead mutant was not able to normalize the elongated mitochondrial phenotype (Figs. 4C, D). Quantification of mitochondrial morphology demonstrated the statistical significance of the rescue effect by WT Omi/HtrA2 (Fig. 4D). Interestingly, the level of mitochondrial ROS could also be normalized upon re-transfection of Omi/HtrA2 WT as well as protease-dead (Fig. 4E), suggesting that the morphological phenotype is not due to increased ROS production. Regulation of mitochondrial morphology appears to depend on Omi/HtrA2 protease activity, whereas mitochondrial ROS production could be related to another function of Omi/HtrA2.

It should be noted that the differences observed between both WT and KO as well as in the vector or WT re-transfectants were not due to or did not lead to alterations in the mitochondrial mass as seen by the equal amounts of both the inner membrane protein ANT and the outer membrane protein VDAC1 (Fig. 4F). These results indicate that loss of Omi/HtrA2 leads to disturbed mitochondrial function, without changes in mitochondria mass, and can be rescued by reintroduction of physiological Omi/HtrA2 protein into the KO background.

### Omi/HtrA2 functionally and physically interacts with the mitochondrial fusion protein OPA1

**Fig. 5:**
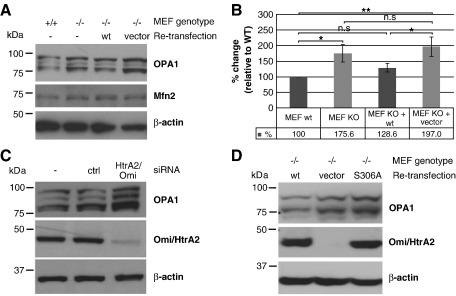
Effects of Omi/HtrA2 on OPA1 protein levels in mouse and human cell lines. (A, B) Omi/HtrA2 WT and KO MEF cells, as well as with WT Omi/HtrA2 and vector stably re-transfected KO cells, were lysed with 1% Triton X-100 in buffer and subjected to immunoblotting. (A) Western blots were probed for the mitochondrial fusion protein OPA1 and Mfn2. β-Actin was used as a loading control. (B) Densitometric analysis of OPA1 band intensities, after 1% Triton X-100 in TNE extraction, in three independent experiments is depicted here (n.s. = not significant, ⁎*p* <   0.05, ⁎⁎*p* <   0.01). Error bars indicate the SD. (C) Effects of Omi/HtrA2 on OPA1 levels were reproduced in HeLa cells. The silencing efficiency as well as the corresponding changes in OPA1 levels was detected in untreated, control or Omi/HtrA2 silenced HeLa cells lysed with 1% Triton X-100 in buffer and subjected to Western blot analysis using antibodies against OPA1, Omi/HtrA2 and β-actin as a loading control. (D) Omi/HtrA2 KO and WT MEF cells were stably re-transfected with either control vector, WT or protease mutant [S306A]Omi/HtrA2, and the cells were lysed with 1% Triton X-100 buffer and subjected to Western blot analysis using antibodies against OPA1 , Omi/HtrA2 and β-actin as a loading control.

Our results suggest an involvement of Omi/HtrA2 in the regulation of mitochondrial morphology. The latter is controlled by mediators of mitochondrial fission, such as Drp1 and Fis1, and by mediators of mitochondrial fusion, such as OPA1 and mitofusins [14]. Interestingly, we discovered a selective increase of OPA1 protein levels, but not in another mitochondrial fusion protein (Mfn2) in Triton X-100 extracts prepared from Omi/HtrA2 KO MEF, when compared to controls (Fig. 5A). Densitometric quantification of the effect indicates a significant increase in mild detergent extractable OPA1 levels in KO cells (Fig. 5B), without affecting the relative amounts of each isoform. Consistently, we found that re-transfection of KO cells with WT Omi/HtrA2 not only rescued the observed mitochondrial phenotype (Figs. 4A, D), but also reverted the increased OPA1 levels to that seen in WT controls (Figs. 5A, B). This effect was confirmed in HeLa cells treated with Omi/HtrA2 siRNA. These cells also displayed elevated mild detergent-extractable OPA1 levels compared to control siRNA treated cells (Fig. 5C). Importantly, similar to the elongated mitochondrial phenotype, the altered OPA1 protein levels could not be rescued by re-transfection of the protease dead variant of Omi/HtrA2 into the Omi/HtrA2 KO MEF cells (Fig. 5D).

**Fig. 6:**
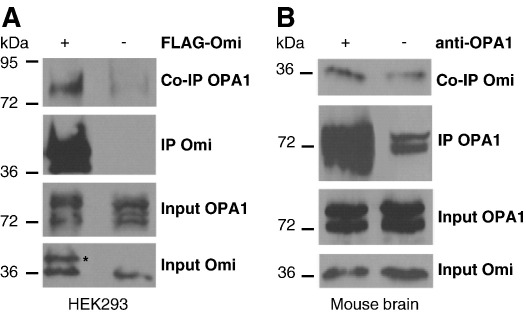
Omi/HtrA2 physically interacts with OPA1. (A) HEK293 cells were stably transfected with FLAG-tagged Omi/HtrA2 (+) while untransfected cells (−) were used as controls. Lysates were subjected to Western blot analysis directly (inputs) or after incubation overnight with anti-FLAG coupled agarose. Immunoblots were probed with anti-OPA1 and anti-Omi/HtrA2 as indicated. The asterisk marks the recombinant form of Omi/HtrA2. The input sample was taken before adding the agarose. (B) Co-immunoprecipitation of endogenous Omi/HtrA2 and OPA1 in lysates from C57/Bl6 mouse brain. Lysates were subjected to Western blot analysis directly (inputs) or after incubation overnight with either protein G agarose alone (−) or with anti-OPA1 bound to protein G agarose (+). Immunoblots were probed with anti-OPA1 and anti-Omi/HtrA2 as indicated.

Since these findings were indicative of a functional relation between Omi/HtrA2 and OPA1, we investigated a physical interaction between these two proteins. We observed a direct interaction of overexpressed FLAG-tagged Omi/HtrA2 protein with endogenous OPA1 protein in co-immunoprecipitation experiments using HEK293 cells (Fig. 6A). Moreover, endogenous Omi/HtrA2 was also found to co-immunoprecipitate with OPA1 in lysates from mouse brain (Fig. 6B). Although some OPA1 unspecifically binds to the agarose (and consequently pulls down some Omi/HtrA2), we can detect a clear enrichment of Omi/HtrA2 in the OPA1 immunoprecipitates compared to the control without OPA1 antibody.

### Omi/HtrA2 affects a Triton X-100 extractable and proteinase K labile pool of OPA1

**Fig. 7:**
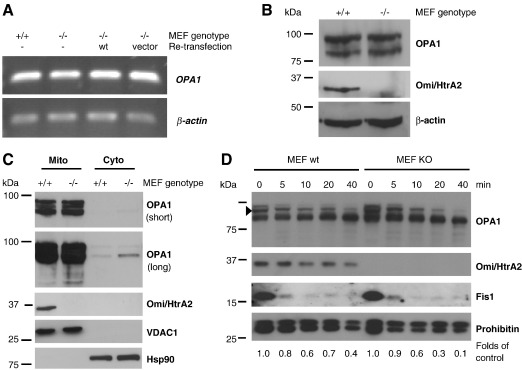
Differences in OPA1 levels are due to protein accessibility. (A) Semiquantitative RT-PCR was performed on mRNA isolated from Omi/HtrA2 WT or KO and re-transfected MEF cells. There are no differences in the *OPA1* mRNA levels (upper panel). Amplification of *β-actin* served as loading control (lower panel). (B) For analysis of the full cell content, 150,000 control and Omi/HtrA2 KO MEF cells were harvested, lysed directly in Laemmli SDS-PAGE sample buffer and analyzed by Western blot. Immunoblots were probed with anti-OPA1 (short and long exposure) and anti-Omi/HtrA2 and β-actin was used as a loading control. (C) Mitochondrial and cytosolic fractions prepared from Omi/HtrA2 WT and KO cells were subjected to Western blotting and analyzed for localization of both OPA1 and Omi/HtrA2. The purity of the cytosolic (Cyto) and mitochondrial (Mito) fractions was assessed by probing for Hsp90 and VDAC1, respectively. (D) Mitochondria isolated from control (left lanes) or Omi/HtrA2 KO (right lanes) MEF cells were subjected to proteinase K digestion for indicated time points. Western blots were prepared and probed with anti-OPA1 and anti-Omi/HtrA2 as indicated. The outer membrane associated protein Fis1 was rapidly degraded whereas the inner membrane protein prohibitin was shielded. The higher molecular weight OPA1 band densitometrically analyzed (arrow head) is indicated and the fold change compared to untreated (time point 0) is shown below the Western blot (D).

The effects of Omi/HtrA2 on OPA1 protein levels appeared to occur at the post-translational (protein) level. We found no difference in the mRNA levels of OPA1 between Omi/HtrA2 KO MEF and wild-type controls, as determined by semiquantitative RT-PCR of all isoforms of OPA1 (Fig. 7A).

Interestingly, loss of Omi/HtrA2 influenced preferentially a Triton X-100 soluble pool of OPA1. Under harsher detergent conditions, such as using SDS no difference between Omi/HtrA2 KO MEF cells and WT controls was observed (Fig. 7B, and results not shown). These findings suggest that Omi/HtrA2 influences the extractibility of OPA1 that may be related to the submitochondrial localization. We therefore performed limited proteolysis experiments on isolated mitochondria. This can be used to indirectly investigate the localization of proteins within the mitochondria as the proteinase K digestion consecutively digests mitochondrial proteins starting from the outer membrane. Mitochondrial fractions were derived from Omi/HtrA2 KO and control MEF cells and the relative purity of the subcellular fractions was verified by probing for VDAC1 and Hsp90, respectively (Fig. 7C). Interestingly, the accumulated longer OPA1 isoforms in the Omi/HtrA2 KO cells were degraded more rapidly (Fig. 7D). Further densitometric analysis of the higher molecular weight OPA1 band (marked with an arrow head in Fig. 7D) and the fold change compared to the untreated controls (time point 0; Fig. 7D), clearly indicates that the higher molecular weight band of OPA1 is more rapidly degraded.

Finally, we noted an increase of the small cytosolic pool of OPA1 in the Omi/HtrA2 KO MEF cells in longer exposures (Fig. 7C). Release of OPA1 has been described upon disruption of OPA1 engagement in cristae junctions [27,28]. We propose that Omi/HtrA2 is involved in OPA1 cristae junction complex maintenance, as Omi/HtrA2 deficient cells show altered mitochondrial ultrastructure and more soluble OPA1 within mitochondria and cytosol. Some of the PK-labile OPA1 could be engaged in mitochondrial fusion, accounting for the observed mitochondrial elongation in Omi/HtrA2 deficient cells, and the increase in soluble OPA1 release into the cytosol may promote a pre-apoptotic state in Omi/HtrA2 KO MEF cells, consistent with the presence of some activated caspase-3 under basal conditions (data not shown).

## Discussion

Loss of Omi/HtrA2 function has been linked to neurodegeneration in two different neurological disorders, namely Huntington's and Parkinson's disease. Mutations in the Omi/HtrA2 gene that reduced the serine protease activity *in vitro* were found in PD patients and genetic variants that may modulate the expression levels or the stability of Omi/HtrA2 were described in some PD populations [9,10]. Although the role of some of these genetic variants in Omi/HtrA2 is still debated [29,30], a potential contribution of Omi/HtrA2 to PD pathogenesis is underscored by its presence in characteristic intraneuronal protein aggregates in PD that not only contain α-synuclein protein but also show a specific co-staining with Omi/HtrA2 not observed in other neurodegenerative disorders [9,31].

Here we present evidence that Omi/HtrA2 is a critical factor for the maintenance of mitochondrial integrity and that loss of Omi/HtrA2 function leads to dysfunctional mitochondria. Indeed, we found that Omi/HtrA2 KO MEFs display an elongated mitochondrial phenotype and impaired mitochondrial function as indicated by increased ROS levels and reduced mitochondrial membrane potential. Interestingly, the increased ROS levels, unlike the morphology do not seem dependent on protease function, suggesting that these are two separate events but both dependent on the presence of Omi/HtrA2. This also suggests that the increased ROS levels are not the cause of the elongated mitochondria seen in cells devoid of Omi/HtrA2.

Our present data indicate that loss of Omi/HtrA2 function leads to changes in mitochondrial morphology by modulation of the interacting fusion protein OPA1. Thereby our study provides first mechanistic insight into the physiological role of Omi/HtrA2 in the regulation of mitochondrial dynamics and maintenance of mitochondrial homeostasis and supports the pathogenic relevance of loss of Omi/HtrA2 function in neurodegeneration. The observed interaction of Omi/HtrA2 with OPA1 extends this link to the molecular level and reveals a novel role of Omi/HtrA2 in balancing mitochondrial fusion-fission dynamics. Importantly, the role of Omi/HtrA2 in the regulation of mitochondrial morphology and the effects of the loss of the protein are not transient as we observe the same mitochondrial phenotype in both a model of genetic ablation of Omi/HtrA2, as well as upon transient knock down of the protein. This contrasts with the situation where PINK1 is deleted, where the paradigm applied for analysis, acute versus chronic, seems crucial for observing changes in mitochondrial morphology [36]. Furthermore, we showed that the role of Omi/HtrA2 as a modulator of mitochondrial morphology is highly conserved as these effects are independent of cell type and species. In addition, we could clearly show that this is dependent on the serine protease activity of Omi/HtrA2. Recently altered mitochondrial dynamics were linked to neurodegeneration in PD by the functional characterization of proteins mutated in familial forms of PD (PINK1 and Parkin). Loss of PINK1 or Parkin function was related to dysregulation of the mitochondrial fusion-fission machinery with PINK1 acting upstream of Parkin [21,32]. Omi/HtrA2 has also been found to be regulated by PINK1 [25], and recent genetic studies in *Drosophila* suggest that Omi/HtrA2 acts downstream of PINK1 but parallel to Parkin in maintaining mitochondrial integrity in *Drosophila* [33,34]. It was shown that the loss of Omi/HtrA2 leads to a weak mitochondrial phenotype in *Drosophila*, with only mild alterations in mitochondrial integrity upon aging that correlated with progressive motor deficits in these animals [34], which was not described in an independent model [35]. Here we have studied the effects on mitochondrial morphology in Omi/HtrA2 silenced *Drosophila* cells, and found a similar mitochondrial elongation as in mammalian cells.

In our study, loss of Omi/HtrA2 function was not only related to elongated mitochondrial morphology seen by light microscopy (Fig. 2), but also with differential extractability of the interacting protein OPA1. Remarkably, Omi/HtrA2 appears to influence preferentially a Triton-soluble, proteinase K labile pool of OPA1 protein (Figs. 5 and 7). Furthermore, as OPA1 is known to play a role in maintenance of the inner membrane structure [27,37], and we see a loss of cristae and cristae junction structures in the KO MEFs (Fig. 2), it is tempting to speculate that the easier extractable material observed represents OPA1 not engaged in the tight complex that seals mitochondrial cristae junctions. Disruption of cristae junctions leads to some cytosolic release of OPA1 [27,28], and indeed we observe an elevation of a small cytosolic pool of OPA1 specifically in Omi/HtrA2 KO MEF cells. We therefore speculate that Omi/HtrA2 might influence the mitochondrial inner membrane structures and fusion by modulating the localization of OPA1 to either the cristae junctions or the potentially more easily extractable fusion complex. This might be mediated by the physical interaction of Omi/HtrA2 with OPA1, as indicated by co-immunoprecipitation experiments.

In summary, we identified for the first time a direct interaction of Omi/HtrA2 with OPA1, a regulatory component of the mitochondrial fusion machinery, and demonstrate consequences of loss of Omi/HtrA2 function on mitochondrial dynamics and integrity that are relevant for different neurodegenerative disorders like Parkinson's and Huntington's disease.
